# Effect of Chemical Treatments on Flax Fibre Reinforced Polypropylene Composites on Tensile and Dome Forming Behaviour

**DOI:** 10.3390/ijms16036202

**Published:** 2015-03-17

**Authors:** Wentian Wang, Adrian Lowe, Shankar Kalyanasundaram

**Affiliations:** Research School of Engineering, Australian National University, North Road, Canberra 0200, Australia; E-Mails: adrian.lowe@anu.edu.au (A.L.); shankar.kalyanasundaram@anu.edu.au (S.K.)

**Keywords:** anisotropic materials, polymer-matrix composites, mechanical properties, wettability, stamp forming

## Abstract

Tensile tests were performed on two different natural fibre composites (same constituent material, similar fibre fraction and thickness but different weave structure) to determine changes in mechanical properties caused by various aqueous chemical treatments and whether any permanent changes remain on drying. Scanning electronic microscopic examinations suggested that flax fibres and the flax/polypropylene interface were affected by the treatments resulting in tensile property variations. The ductility of natural fibre composites was improved significantly under wet condition and mechanical properties (elongation-to-failure, stiffness and strength) can almost retain back to pre-treated levels when dried from wet condition. Preheating is usually required to improve the formability of material in rapid forming, and the chemical treatments performed in this study were far more effective than preheating. The major breakthrough in improving the formability of natural fibre composites can aid in rapid forming of this class of material system.

## 1. Introduction

Recently, natural fibre reinforced composites have gained increasing acceptance in the field of engineering, through research and commercial interests [1,2,3]. Due to the low density of natural fibres, composites reinforced with natural fibres can result in lightweight structures that appeal to the automotive industry amongst others [4]. Compared to conventional monolithic metal, manufacturing automobile parts with such composite systems is able to significantly increase the fuel efficiency of vehicles leading to benefits such as reduced emissions. In addition to this, biodegradability factors allow for more end-of-life options to be considered. However, the major drawback with natural fibre composites is their low thermal resistance, which is a particular issue if temperature is used to improve the processability of these systems [5].

Stamp forming was developed as a rapid processing technology for converting sheet metal into a variety of thin automotive components and has been successfully used to form lightweight composite materials, [6]. Davey *et al.* [7] formed a carbon fibre reinforced polyether ether ketone (PEEK) composite through stamp forming at room temperature. His work suggested that the variations in allowable strain in the carbon fibres affected the locations of failure regions within the dome structure. Compston [8] compared the surface strain for stamp formed aluminium and an aluminium-Curv (self-reinforced polypropylene composite) laminate. It was found that the aluminium-Curv exhibits lower surface strain and is more resistant to the change of tool radii. Given the results from these studies, it should be possible to manufacture panels from natural fibre composites using existing stamp forming facilities at little extra cost, which also exhibit favourable forming characteristics.

Heat treatment is known to be effective in improving the formability of polypropylene composites in parts forming [9,10,11,12,13]. However, the poor thermal resistance of natural fibres results in their thermal degradation during forming operations at relatively low temperatures. Therefore, there is an acknowledged need for an alternative way to modify natural fibre-based composites to improve their formability, and chemical treatments on fabrics before consolidation are being seen as an effective alternative [14,15,16,17,18,19,20]. For instance, Van De Velde [16] showed that treating flax/polypropylene composite with the Maleic Acid (M.A) solution, chemical entanglements form within a maleic anhydride/polypropylene copolymer and that this physical link improves load transfer between fibres. Espert *et al.* [19] found that there is degradation of the fibre/matrix interface when a cellulose/polypropylene composite is treated with water. It has been noted that little research has been performed on investigating the effect of the chemical treatment on pre-consolidated natural fibre composite. This is important knowledge to understand especially when there is a high level of porosity in natural fibre composites caused during the hot consolidation process. This paper aims to determine the effect of various chemical treatments on the tensile properties and formability of pre-consolidated flax fibre reinforced polypropylene composites both in the saturated and redried condition. A scanning electron microscope (SEM) examination was carried out on the tensile failure surfaces to help explain any observed tensile property variations. Specimens were formed in a dome structure to study two major forming modes of stretching and drawing encountered in dome forming operations, and formability was quantified through observation of the depth-to-failure during dome forming in this study.

## 2. Results and Discussion

### 2.1. Behaviour of the Composites during Moisture Ingress and Moisture Egress Processes

**Figure 1:**
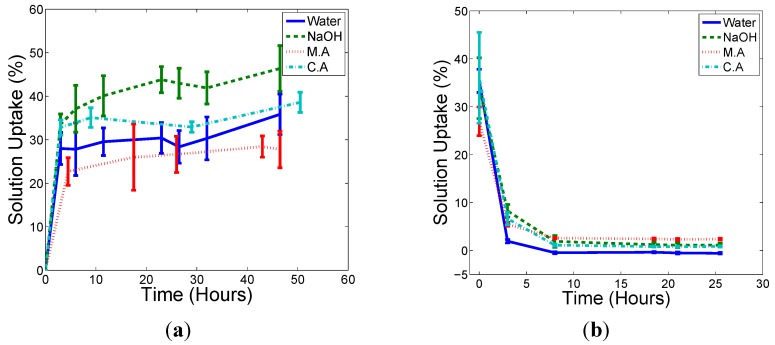
The rate of solution uptake (**a**); and subsequent moisture egress from saturation (**b**) for the CNFC rectangular samples.

**Figure 2:**
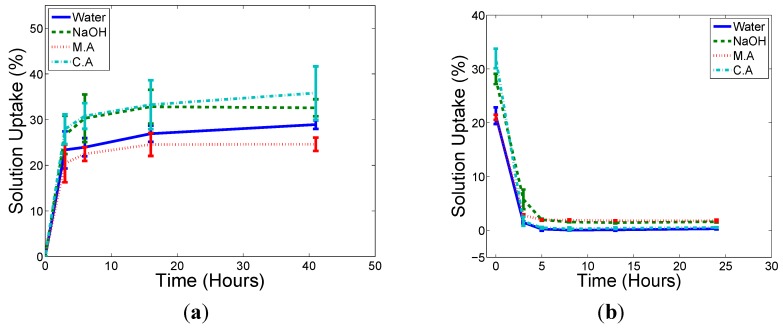
The rate of solution uptake (**a**); and subsequent moisture egress from saturation (**b**) for the NFC rectangular samples.

Figure 1 and Figure 2 show the solution uptake (to saturation) and solution removal (subsequently dried) curves for the chopped natural fibre composite (CNFC) and the continuous natural fibre composite (NFC), respectively. Samples are of rectangular dimensions 15 mm by 150 mm. Solution uptake (Δm(t) in %) is defined through the following equation:

[Δm(t) = (W_t_ − W_o_)/W_o_ × 100%] (1)

where W_o_ is the original weight of the specimen and W_t_ is the weight of the specimen at time t. The samples were weighed after removal from the liquid reservoirs followed by immediate shaking to remove surface moisture. No surface moisture was detected during this weighting process and the samples were returned to the reservoirs once a stable weight reading had been recorded. Sample sizes of 5 were used for this study.

The samples all reached the maximum amount of chemical solution uptake in a similar, short amount of time (taking a matter of hours), which is much quicker than other systems, such as those reported by Wang [20], who showed that a HDPE/rice husk composites system (with volume fraction ranging from 40% to 60%) with a dimension of 12 cm × 12 cm × 2.4 cm reach saturation after over 270 h of immersion in water. One reason for this speed may be the high porosity observed in these samples. The M.A solution has the highest solution density and both composites have the lowest amount of solution uptake in M.A treatment compared to other treatments. No gaseous by-products were observed in any system.

A small amount of residual weight remained in dried samples that were treated with NaOH and M.A solutions. This is not surprising given that both of these solutions were mixed from dry chemical powders and so would most likely remain in the sample after any water has been dried off. In addition, it was noted that the dye used to colour the fibres in the CNFC samples (mostly blue and red) had been removed during the saturation process but this was thought not to affect the weight change measurements or to contribute to any chemical reaction.

**Figure 3:**
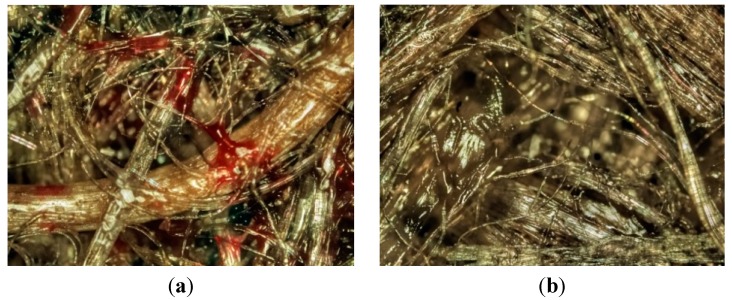

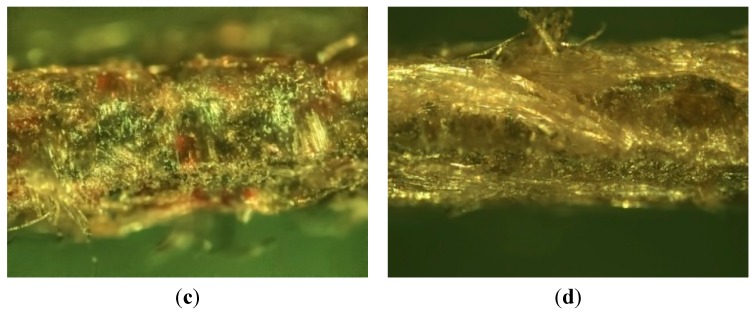
Optical micrographs of CNFC composite (**a**,**c**) and the NFC composite (**b**,**d**). The images (**a**) and (**b**) are of the composite surface whilst the images (**c**) and (**d**) are of the centre cross-section region.

Figure 3 illustrates that both composites have significant porosity issues throughout the sample thickness, suggesting consolidation issues such as gaseous emission during the manufacturing process. Previous research, e.g., [21], demonstrates that synthetic fibre composites can attain porosity levels of less than 1% if processed accurately. Such low values are not yet possible in natural fibre/thermoplastic composites as no processing technology can compensate for the very low wettability properties seen in these materials that can also lead to poor interfacial quality [4]. The current study estimates that porosity levels in excess of 25% occur, with the higher values seen in the NFC materials. This may be the reason for the smaller saturation levels seen in the NFC sample set as the air bubbles may prevent moisture from progressing to the fibre/matrix interface which is where most of the moisture probably sits. Indeed, since polypropylene is hydrophobic, only the flax fibre and the flax-polypropylene interface can absorb the solutions. Because of this, the fibre/matrix interface (and the fibres themselves) should exhibit chemical and physical changes (permanent or not) that will affect mechanical properties, such as tensile behaviour and formability. Similar findings were obtained in previous studies [18,19].

### 2.2. Effect of Chemical Treatment on the Tensile Behaviour of the Composites

#### 2.2.1. Variations on Tensile Properties

**Figure 4:**
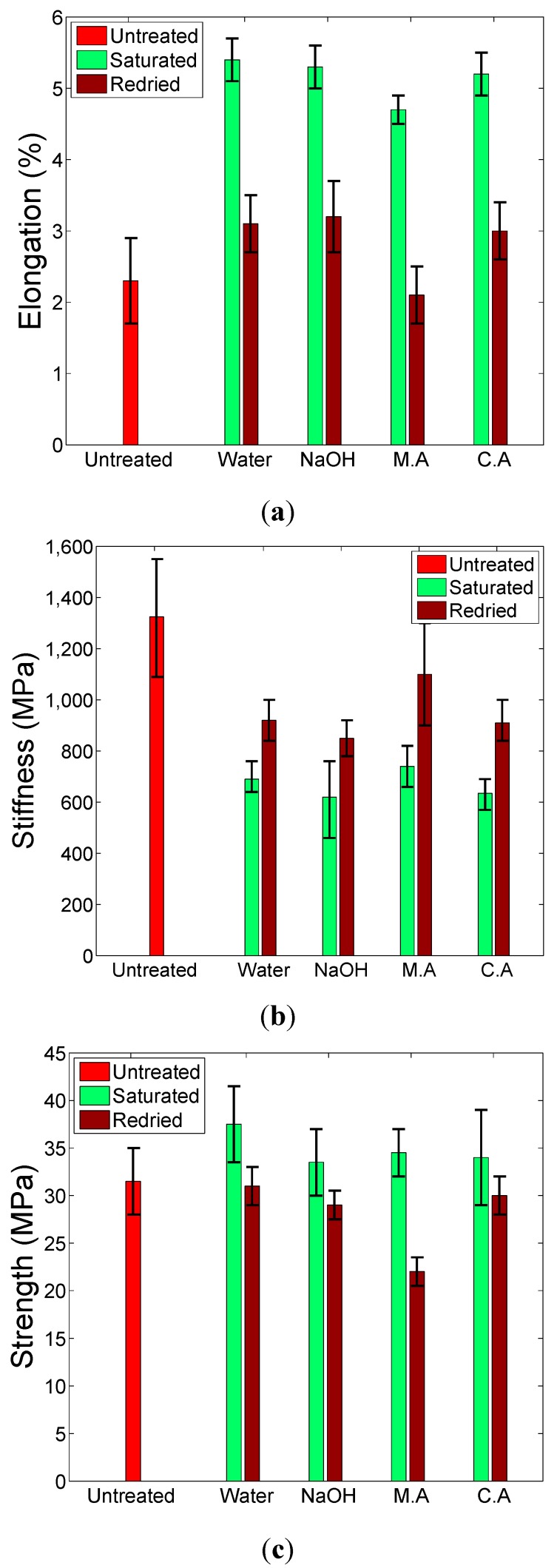
Tensile property data for CNFC samples under various aqueous treatments showing (**a**) elongation; (**b**) stiffness and (**c**) strength.

Rectangular tensile test specimens of 15 mm by 150 mm were produced for tensile tests, generating elongation-to-failure, stiffness and strength data. For the NFC samples, the samples were cut such that fibres were oriented at 0°/90° to the sample axis. Figure 4 shows how the tensile properties for CNFC samples vary with treatment. Tensile stiffness is permanently reduced by the saturation/drying process, whereas elongation-to-fracture is increased significantly when saturated, but largely returns to pre-soaked values when dried suggesting a non-permanent effect. Strength, however, does not seem to be statistically affected by the saturation/drying process, although the maleic anhydride-treated samples may have statistically weakened. The stiffness results suggest that the fibre/matrix interface has been permanently altered. When the samples are saturated, all treatments significantly increase the elongation properties whilst decreasing the stiffness response, suggesting that formability has been improved, without a loss of strength. These observations suggest that it would be very advantageous to form this class of material system in wet condition, especially in designs where stiffness response is of secondary importance. According to Van De Velde [16], treatment with maleic anhydride should improve the interfacial strength between fibre and matrix. However, this effect is not evident in this study, probably because the aqueous and saturated nature of the treatment does not allow for the chemical to form the correct chemical bonds with the constituents. Such treatments are often invoked using dry conditions such as adding anhydride powder to the liquid polymer, or soaking the fibres in an anhydride solution that is then subsequently dried prior to mixing with the polymer.

**Figure 5:**
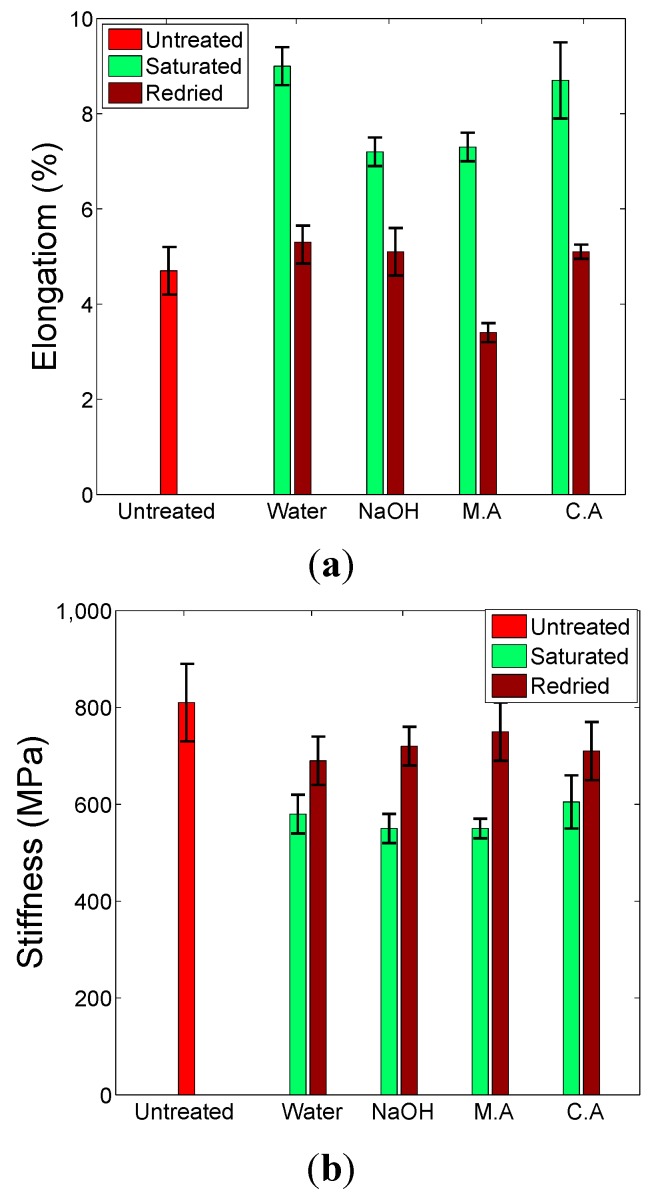

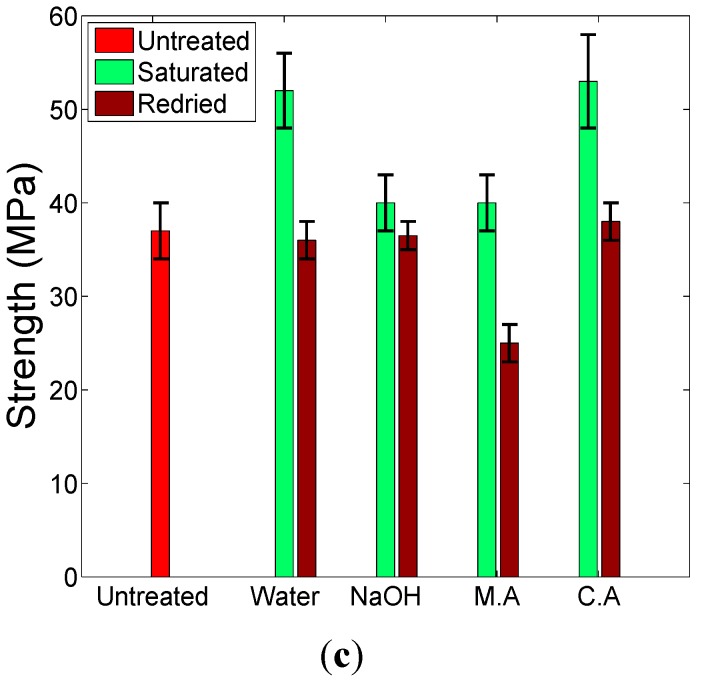
Variations in mechanical properties of the NFC under different chemical treatments obtained through tensile tests, (**a**) elongation; (**b**) stiffness and (**c**) strength.

The corresponding mechanical responses seen in the NFC material are detailed in Figure 5. Compared to the CNFC sample set, there are a few slight differences. Firstly, the magnitude of the irreversible change in stiffness seems much less severe in the NFC materials, particularly when redried; and secondly, the strength values seem to increase when soaked in pure water or cloudy ammonia solution and return back to original values when redried; Thirdly, as with the previous sample set, the maleic anhydride-treated material appears to be permanently weaker than the others. These differences can be attributed to the difference in reinforcement morphology. In the CNFC samples, the fibres are randomly oriented whereas in the NFC samples, the fibres are in the form of a 2 × 2 twill structure. Therefore, it is anticipated that in the NFC samples, the fibres are more restricted in their possible movement during tensile testing and will only travel in the weave directions. In woven composites, the failure mechanisms are more fibre-dominated than in their random fibre equivalents and so if there are any changes happening within the cellulosic fibres during soaking, they would be more apparent in the NFC samples. Overall, chemical changes that cause tensile property variations are strongest in solutions that do not contain relatively bulky solute substances (maleic anhydride or sodium hydroxide). Such solutions may find it harder to permeate the material compared to pure water or ammonia, and thus find it harder to completely hydrolyse the interfacial region. In addition, plasticization of the flax fibres is a known phenomenon as explained in [22] with pure water and ammonia treatments—which are often used as plasticizers for wood.

#### 2.2.2. SEM Examinations on Fractured Surfaces

**Figure 6:**
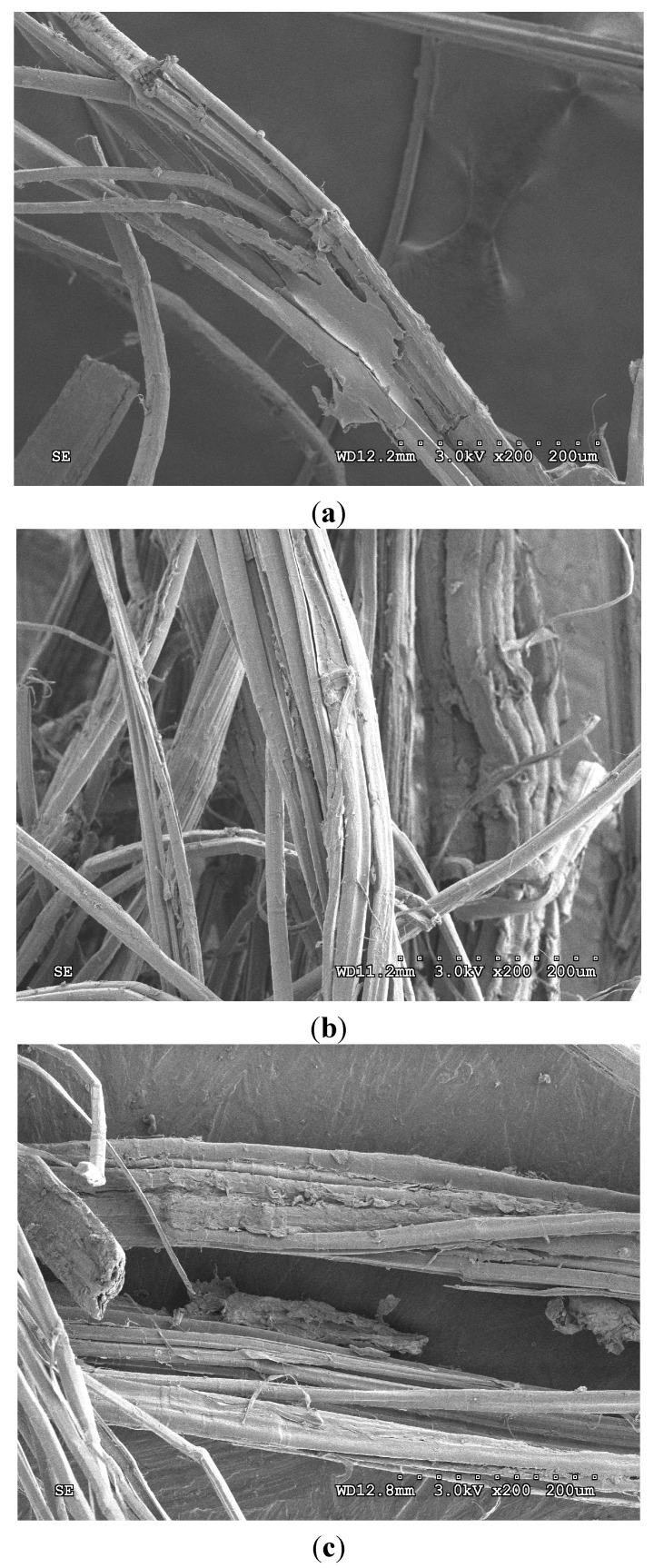
Electron microscope images of CNFC showing (**a**) untreated; (**b**) saturated and (**c**) redried samples that have been fractured under tensile tests.

Figure 6 shows the SEM images of CNFC fracture surfaces from samples tested under different conditions. For the untreated and dried specimens, the fractured flax fibre bundles are coated with polypropylene matrix and flax fibre bundles cannot be easily differentiated from the polypropylene matrix. This is a statistically consistent observation suggesting a relatively strong interface between the flax fibre and the polypropylene matrix [23]. Clean fibre surfaces are seen in the saturated sample, that is, there appears to be no post-fractured resin regions adhered to the fibre surface and this is indicative of a much weaker fibre-matrix interface when hydrolysed. It has been reported [19] that when solutions are absorbed by the fibre/matrix interface, the cellulosic fibres tend to swell which favours fibre debonding and weakens the fibre/matrix interface leading to tensile property variations. This would appear true for the current study, as the interfacial properties (stiffness and elongation) are significantly changed when wet. In addition, the strength of the materials appear to remain largely inert to the saturation and redrying process and no significant changes to fibre fracture morphology was detected during the fractographic study. Therefore, if the cellulosic fibres swell during the saturation process, they remain chemically unchanged, whilst initiating changes at the interfacial region. On drying, the fibre-matrix integrity seems to return, but there is a permanent reduction in composite stiffness. This could suggest the presence of an interphase region that remains permanently weakened from the saturation process. Thus when dried, the samples retain strength (no fibre changes observed), and regain their interfacial characteristics (as per the SEM observations) whilst maintaining a reduced stiffness through mechanical weakness in the interphase region, which may be pre-existing, or may have formed as a result of the saturation process. Mishra [24] had a similar finding that the stiffness of natural fibre composite is not fully recovered after drying the samples, and this permanent damage to the fibre is attributed to the interfacial degradation and structure breakdown of the cell wall in natural fibres. A possible exception to these trends is the maleic anhydride-treated material, where strength seems to be permanently reduced once the samples are dried. This could be due to chemical ingress of the anhydride solution into the cellulosic fibres which when dried, cause chemical changes that permanently weaken the fibres, although this has not been detected during fractographic examination.

Given the nature of the reinforcement, the fibre fracture mechanisms will dominate more in the woven twill (NFC) samples than in the random fibre (CNFC) samples and so the changes observed in the NFC samples could be due to some fibre plasticization effect that is most evident in pure water and ammonia treatments, and this effect may dominate over the interphase and interface-based mechanisms seen in the CNFC samples. It is likely therefore, that both mechanisms exist in the two materials, with one clearly dominating the other.

### 2.3. Effect of Water Treatment on Dome Formability

Given the large property differences seen in both materials between wet and dry samples, dome forming tests were performed to see if corresponding changes in formability were realised. All dome forming experiments conducted in this study are in displacement control and as it has been shown that pure water and cloudy ammonia produce the greatest improvement on the failure elongation of both composites in the saturated condition, samples treated in pure water were chosen for this part of the study.

**Figure 7:**
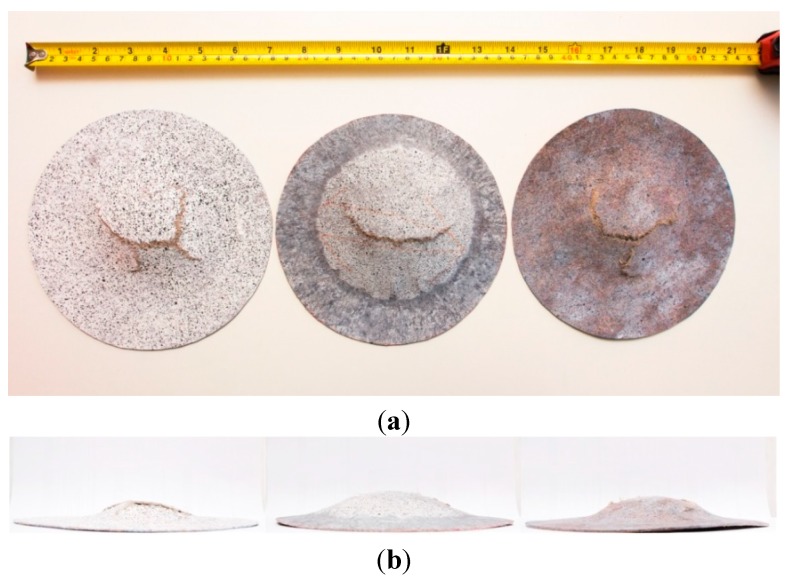
Images of the untreated, saturated and dried CNFC (from left to right) after stamp forming experiments from, (**a**) top view; and (**b**) front view.

**Figure 8:**
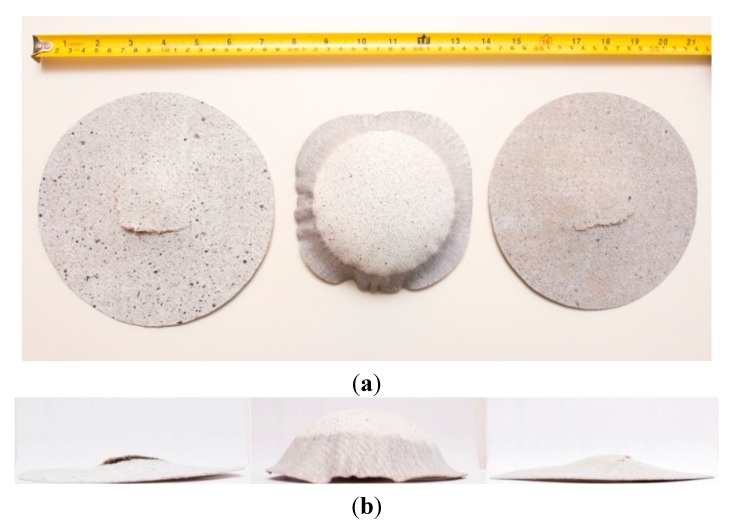
Images of the untreated, saturated and dried NFC (from left to right) after stamp forming experiments from, (**a**) top view; and (**b**) front view.

Figure 7 and Figure 8 show the top view and front view of the CNFC and NFC composite domes respectively. The middle sample in each figure is from a saturated disc whilst the untreated and redried samples are to the left and right, respectively. In this study, the main focus of the dome forming experiments is the failure depth which is defined as the vertical displacement of the pole at failure. Materials which are able to form to a large depth whilst maintaining mechanical integrity lend themselves to making parts with a high level of complexity. Such materials would be very useful and competitive in the manufacturing sector. The observations in failure depth highlight the need of the water treatment when the composite is formed to parts with high level of complexity.

**Figure 9:**
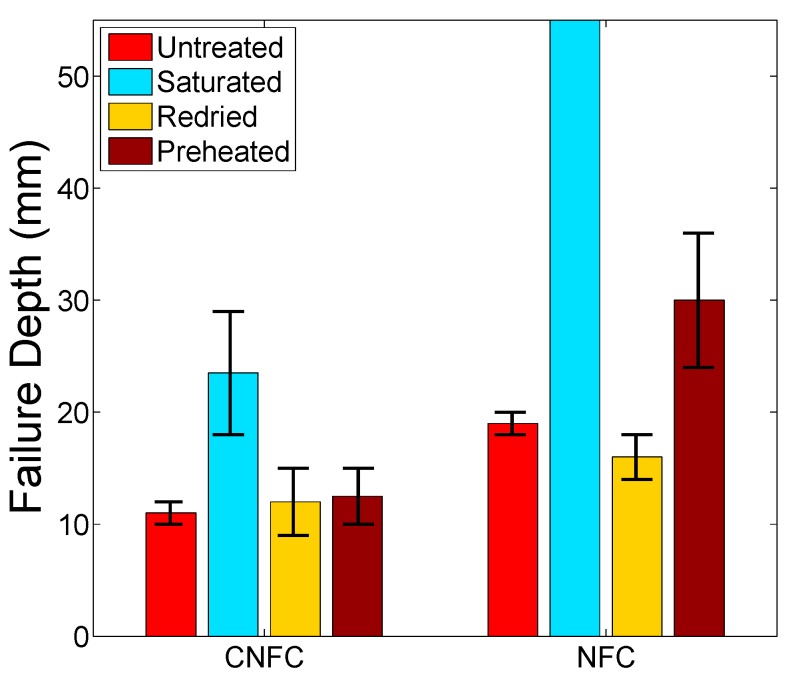
A comparison of the forming depth in stamp forming between the CNFC and the NFC.

Figure 9 shows that both samples became increasingly formable under saturated conditions and samples that were redried had identical formability characteristics to the untreated samples. This is in agreement with the previous tensile test study, given that elongation-to-failure and strength are very influential in the forming process. The temporary fibre plasticization effect previously discussed has a clear effect during forming tests, as failure data for the saturated NFC sample could not be achieved as the domes frequently reached the maximum punch distance of 55 mm without failing. This is a very favourable observation, especially as mechanical properties largely return to untreated levels when redried. For the flax/polypropylene composite, greater forming depths at high forming temperatures are permitted by developing matrix shear deformation during forming [13]. However, Wentian *et al.* [13] found that flax/polypropylene composites start degrading when formed at a temperature close to the melting temperature of polypropylene, and the study also found the optimal temperature window for this class of material system. In order to compare the effect of these two treatments on natural fibre composites, both composites were preheated to the optimal temperature window and then formed in this study. It is found that the water-treated flax/polypropylene composites are able to exhibit an almost doubled forming depth compared to that of preheated equivalent samples. In addition to the fact that less amount of energy input is required during the treatment, the water treatment can be more effective than the heat treatment in terms of improving the formability of natural fibre composites in dome forming. 

## 3. Experimental Section

Two classes of flax fibre reinforced polypropylene (pp) composites were analysed in this study. A pre-consolidated chopped flax fibre reinforced polypropylene composite, FibriBoard™, was manufactured by EcoTechnilin, Kimbolton, UK. A continuous flax fibre reinforced polypropylene fabric, Biotex™, was manufactured by Composite Evolution, Chesterfield, UK and then consolidated by the Xiafei factory located in Jiayong junctions, Dongguan, Guangdong, China. The chopped natural fibre composite has a random distribution of short flax fibres and the fibre bundles in the continuous natural fibre composite are woven in a 2 × 2 twill structure. Both composites have the same constituent material, a similar fibre weight fraction (50%) and thickness (1 mm). It was noted that all samples appeared to possess a higher than expected level of surface porosity, and that this porosity seemed to continue through the sample thickness. It was assumed that this porosity was caused by the evolution of gaseous products during the hot consolidation process—a common issue with natural fibre composites. Distilled water, a 0.5 M sodium hydroxide solution (NaOH), a 12% weight percentage M.A solution and a cloudy ammonia (C.A) solution with 20 g/L of ammonia were selected to determine the effect of chemical treatments on the physical and tensile properties of the samples. To determine the saturation characteristics in each solution at ambient temperature, samples were fully immersed in plastic containers filled with chemical solutions up to a depth of 35 mm. A circular hole was drilled in the middle of the lid and sealed with a piece of flexible parafilm to create a gas bleeding mechanism and ensure safe testing conditions. 

Three sample conditions were tested: untreated, saturated and dried. Untreated samples were cut from the as-received material with no chemical treatment; saturated testing was performed on samples that had reached a consistent weight gain; and dried testing was performed on samples that had been saturated, and then subsequently dried. All tensile tests were conducted using an Instron™ 4505 testing frame, manufactured by Instron, Melbourne, Australia, in atmospheric conditions. Specimens were tested at a rate of 5 mm/min with failure being defined as when the applied load had dropped to 40% of its original value. To determine the driving mechanisms behind the variations in mechanical properties observed, failure regions of the tested samples were lightly platinum coated for Scanning Electron Microscope (SEM) examinations through a Hitachi 4300 Field Emission Electron Microscope, manufactured by Hitachi, Sydney, Australia. Samples for the forming tests were circular and cut to a diameter of 180 mm through a mechanical scissor and were placed in a 30 ton customised press machine prior to forming using a dome punch. All samples were formed under a blank holder force of 7 kN and a feed rate of 20 mm/s.

## 4. Conclusions

This study reports how various aqueous treatments affect the formability of polypropylene reinforced with chopped random or continuous woven flax fibres. Moisture uptake was very rapid and tensile tests showed that when saturated, the chopped random fibre material exhibits a significantly and temporarily increased elongation-to-failure, a permanently reduced stiffness and unchanged strength. These observations suggest that there may be a temporary plasticization effect in the interface region (especially in the pure water and the cloudy ammonia-treated materials) that causes elongation to increase. Fibre-based mechanisms are more dominant in the woven composite materials where similar elongation behaviour was observed (temporary), and the reduction in stiffness was also similar (permanent), but less dominant. Composite strength was seen to increase significantly in pure water and cloudy ammonia treatments, probably through fibre plasticization leading to stronger fibres. The water and cloudy ammonia solutions were far more effective in inducing property changes than the maleic anhydride solution, suggesting that the molecular nature of the latter inhibited both moisture ingress and subsequent plasticization mechanisms. Dome forming experiments showed that the water treatment can significantly improve the formability of composites, such that composites became significantly more formable (doubled failure depth) when tested in wet condition. This work also demonstrates that the treatment reported here can be more effective than preheating, which is the conventionally applied method in material forming.
